# The treatment with trandolapril and losartan attenuates pressure and volume overload alternations of cardiac connexin-43 and extracellular matrix in Ren-2 transgenic rats

**DOI:** 10.1038/s41598-023-48259-2

**Published:** 2023-11-27

**Authors:** Matus Sykora, Vojtech Kratky, Ludek Cervenka, Libor Kopkan, Narcis Tribulova, Barbara Szeiffova Bacova

**Affiliations:** 1 Centre of Experimental Medicine, Institute for Heart Research, Slovak Academy of Sciences, 841 04 Bratislava, Slovakia; 2 Center for Experimental Medicine, Institute for Clinical and Experimental Medicine, 140 21 Prague, Czech Republic; 3 Department of Nephrology, First Faculty of Medicine, Charles University and General University Hospital in Prague, 128 08 Prague, Czech Republic; 4 Department of Internal Medicine I, Cardiology, University Hospital Olomouc and Palacky University, Olomouc, Czech Republic

**Keywords:** Experimental models of disease, Protein-protein interaction networks

## Abstract

Heart failure (HF) is life-threatening disease due to electro-mechanical dysfunction associated with hemodynamic overload, while alterations of extracellular matrix (ECM) along with perturbed connexin-43 (Cx43) might be key factors involved. We aimed to explore a dual impact of pressure, and volume overload due to aorto-caval fistula (ACF) on Cx43 and ECM as well as effect of renin–angiotensin blockade. Hypertensive Ren-2 transgenic rats (TGR) and normotensive Hannover Sprague–Dawley rats (HSD) that underwent ACF were treated for 15-weeks with trandolapril or losartan. Blood serum and heart tissue samples of the right (RV) and left ventricles (LV) were used for analyses. ACF-HF increased RV, LV and lung mass in HSD and to lesser extent in TGR, while treatment attenuated it and normalized serum ANP, BNP-45 and TBARS. Cx43 protein and its ser368 variant along with PKCε were lower in TGR vs HSD and suppressed in both rat strains due to ACF but prevented more by trandolapril. Pro-hypertrophic PKCδ, collagen I and hydroxyproline were elevated in TGR and increased due to ACF in both rat strains. While SMAD2/3 and MMP2 levels were lower in TGR vs HSD and reduced due to ACF in both strains. Findings point out the strain-related differences in response to volume overload. Disorders of Cx43 and ECM signalling may contribute not only to HF but also to the formation of arrhythmogenic substrate. There is benefit of treatment with trandolapril and losartan indicating their pleiotropic anti-arrhythmic potential. It may provide novel input to therapy.

## Introduction

Heart failure (HF) is the prevalent heart disease of various aetiology facilitating occurrence of malignant arrhythmias, thereby account for substantial morbidity and mortality^1–3^. Failing heart is characterized by mechanical (systolic or diastolic) dysfunction and by electrical instability that aggravates overtime. This promotes sudden cardiac death attributed to ventricular fibrillation (VF)^2,4–7^. The key factors facilitating such life-threatening event are myocardial structural remodelling, hypertrophy, fibrosis along with altered topology and disorders of connexin-43 (Cx43) channels^8–12^. These ensure coupling among cardiomyocytes for transmission of electrical and molecular signals, thereby are essential for synchronized heart function^13^, as suggest our previous and other studies^14–18^. In turn, recent data indicate salutary effects of Cx43 mimetic peptide and Cx43 interacting protein in HF models^19,20^. Nevertheless, myocardial alterations of both Cx43 and extracellular matrix (ECM) due to cardiac volume overload were not rigorously characterized either in normotensive or hypertensive animals.

Ren-2 transgenic rats (TGR) are model of ANG II-dependent hypertension resulting in pressure overload-induced concentric myocardial hypertrophy and ECM deposition over time^21^. Both, pressure and volume overload are clinically relevant models suitable to investigate pathogenesis and to examine efficacy of therapeutic approaches. Notably, there is a knowledge gap referring to myocardial Cx43 and ECM alterations.

Currently, the treatment of HF is based on a combination of drugs aimed at suppressing activation of the sympathetic nervous system, the RAS and agents that positively influence sodium and glucose metabolism^22^. Undoubtedly, the better understanding of yet unravelled mechanisms and processes may promote novel and more effective therapies to improve outcomes^23^.

Coming from our previously published data of echocardiographic parameters in rats with pressure and volume overload, we aimed to test in this experimental model, the response of myocardial Cx43 and ECM, implicated in cardiac remodelling, and arrhythmogenesis^24^. Efficacy of treatment on these molecular pathways with angiotensin converting enzyme inhibitors (ACEi) and angiotensin receptor blockers (ARB) was also subject of our interest.

## Results

### Registered biometric and biochemical characteristic of experimental rats with VO a PO heart failure

In Table 1, compared to HSD rats, the body weight of TGR was significantly higher but reduced due to ACF-induced HF and normalized by treatment with trandolapril or losartan. According to the heart weight to tibia length ratio, there was a mild increase of the heart mass index as well as left and right ventricle mass in TGR versus HSD. ACF-induced HF increased these biometric parameters in both rat strains, while treatment with trandolapril and losartan significantly reduced it in HSD rats and normalized in TGR. Same changes were detected in the lungs weight to tibia length ratio, indicating ACF-induced HF lung congestion. As shown in Table 1, there was no difference in serum ANP levels between normotensive HSD and hypertensive TGR strain. However, ACF-induced HF significantly elevated circulating ANP, in both TGR and HSD rats. Treatment with either trandolapril or losartan mitigated HF-associated elevation of ANP in TGR, while losartan was more effective than trandolapril to reduce elevated serum ANP in HSD rats. On the other side, serum BNP-45 levels were increased in TGR versus HSD. ACF-induced HF increased circulating BNP-45 in HSD rats, while treatment with trandolapril and losartan normalized it in both rat strains. TBARS levels were increased in blood serum of TGR and HSD rats due to ACF-induced HF, while normalized by the treatment with trandolapril in both rat strains and by losartan in HSD rats (Table 1). Besides that, levels of the myocardial tissue TBARS were increased due to ACF-induced HF in both rat strains, but reached statistical significance left ventricle of TGR only (Table 1). These increases were normalized by treatment with either trandolapril or losartan in both heart ventricles (Table 1). In addition, losartan normalized TBARS levels in right ventricle of ACF-affected HSD rats (Table 1). Echocardiography was performed for verification of ACF-induced HF, data recently published^25,26^.

**Table 1:** Registered biometric and biochemical parameters of the experimental rats.

Parameters	HSD	HSD ACF	HSD ACF ACEi	HSD ACF ARB	TGR	TGR ACF	TGR ACF ACEi	TGR ACF ARB
BW (g)	543 ± 37	548 ± 45	537 ± 31	575 ± 56	652 ± 46*	483 ± 46*	606 ± 53^#^	643 ± 38^#^
HW (mg/mm)	40.5 ± 3.3	71.2 ± 5.2*	59.2 ± 4.7*^#^	63.8 ± 4.4*	52.0 ± 4.1*	76.2 ± 3.0*	51.5 ± 8.7^#^	65.2 ± 6.7*^#^
LVW (mg/mm)	18.8 ± 1.9	27.0 ± 2.8*	23.6 ± 3.2*	24. 7 ± 1.3*	22.2 ± 2.7	33.5 ± 3.0*	17.1 ± 3.5^#^	22.4 ± 1.8^#^
RVW (mg/mm)	5.2 ± 0.4	12.9 ± 1.9*	12. ± 1.2*	13.4 ± 1.34*	8.04 ± 1.3*	12.4 ± 0.4*	10.4 ± 1.6	13.5 ± 2.68*
LUW (mg/mm)	46.2 ± 2.2	61.9 ± 12.9*	52.2 ± 5^#^	53.7 ± 6.4^#^	45.6 ± 3.5	65.9 ± 14.1*	52.6 ± 4.9^#^	52.9 ± 4.3^#^
Serum ANP (pg/ml)	2.7 ± 0.3	3.4 ± 0.3*	2.9 ± 0.5	2.3 ± 0.1^#^	2.4 ± 0.2	4.0 ± 0.7*	3.3 ± 0.2^#^	3.3 ± 0.9^#^
Serum BNP-45 (ng/ml)	0.07 ± 0.014	0.15 ± 0.009*	0.06 ± 0.010^#^	0.13 ± 0.006*	0.15 ± 0.010*	0.17 ± 0.015	0.07 ± 0.012^#^	0.08 ± 0.007^#^
Serum TBARS (µmol/µg)	18.7 ± 0.8	25.0 ± 0.8*	19.9 ± 1.9^#^	20.7 ± 1.2^#^	21.3 ± 0.8	24.5 ± 1.1*	20.1 ± 0.9^#^	21.9 ± 2.1
LV TBARS (µmol/µg)	2.4 ± 0.1	2.8 ± 0.1	2.6 ± 0.2	2.6 ± 0.1	2.8 ± 0.1	3.2 ± 0.2*	2.7 ± 0.2^#^	2.7 ± 0.2^#^
RV TBARS (µmol/µg)	2.2 ± 0.2	2.6 ± 0.2	2.3 ± 0.1	2.1 ± 0.1^#^	2.4 ± 0.2	2.9 ± 0.2	2.1 ± 0.2^#^	2.2 ± 0.2^#^
LV HYP (µg/g)	44.3 ± 3.6	56.4 ± 4.3	38.9 ± 2.3	41.3 ± 4.8	49.2 ± 6.8	54.8 ± 6.5	46.6 ± 6.0	43.5 ± 8.0
RV HYP (µg/g)	43.3 ± 1.7	55.2 ± 4.9	34.4 ± 2.7	53.7 ± 5.0	47.7 ± 5.8	72.6 ± 10.9	38.9 ± 4.2	36.3 ± 5.1
Serum MMP-2 (%)	100 ± 8	95 ± 3	83 ± 12	96 ± 4	80 ± 7	65 ± 5	60 ± 12*	78 ± 4
Serum TIMP-2 (pg/ml)	245 ± 39	212 ± 2	341 ± 17*^#^	211 ± 20	171 ± 21	144 ± 20	226 ± 43^#^	134 ± 13

TGR ACF rats suffered with higher incidence of mortality (60%).

*BW* body weight, *HW* heart weight, *LVW* left ventricular weight, *RVW* right ventricular weight, *LUW* lung weight, *TBARS* thiobarbituric acid reactive substances, *MMP-2* activity of matrix metalloproteinase 2, *TIMP-2* Tissue inhibitor of metalloproteinases 2, *ANP* atrial natriuretic peptide, *BNP-45* B-type natriuretic peptide, *HYP* Hydroxyproline, *HSD* normotensive Hannover Sprague–Dawley rats, *TGR* hypertensive heterozygous Ren-2 transgenic (mREN2)27 rats, *ACF* aortocaval fistula, *ACEi* angiotensin-converting enzyme inhibitors, *ARB* angiotensin II receptor blockers.

Results are the mean ± SD (sample size is illustrated in Supplementary Fig. 1), *p < 0.05 vs. HSD/TGR, ^#^p < 0.05 vs. ACF.

### Microscopic evaluation of myocardial tissue and quantitative image analysis

**Figure 1:**
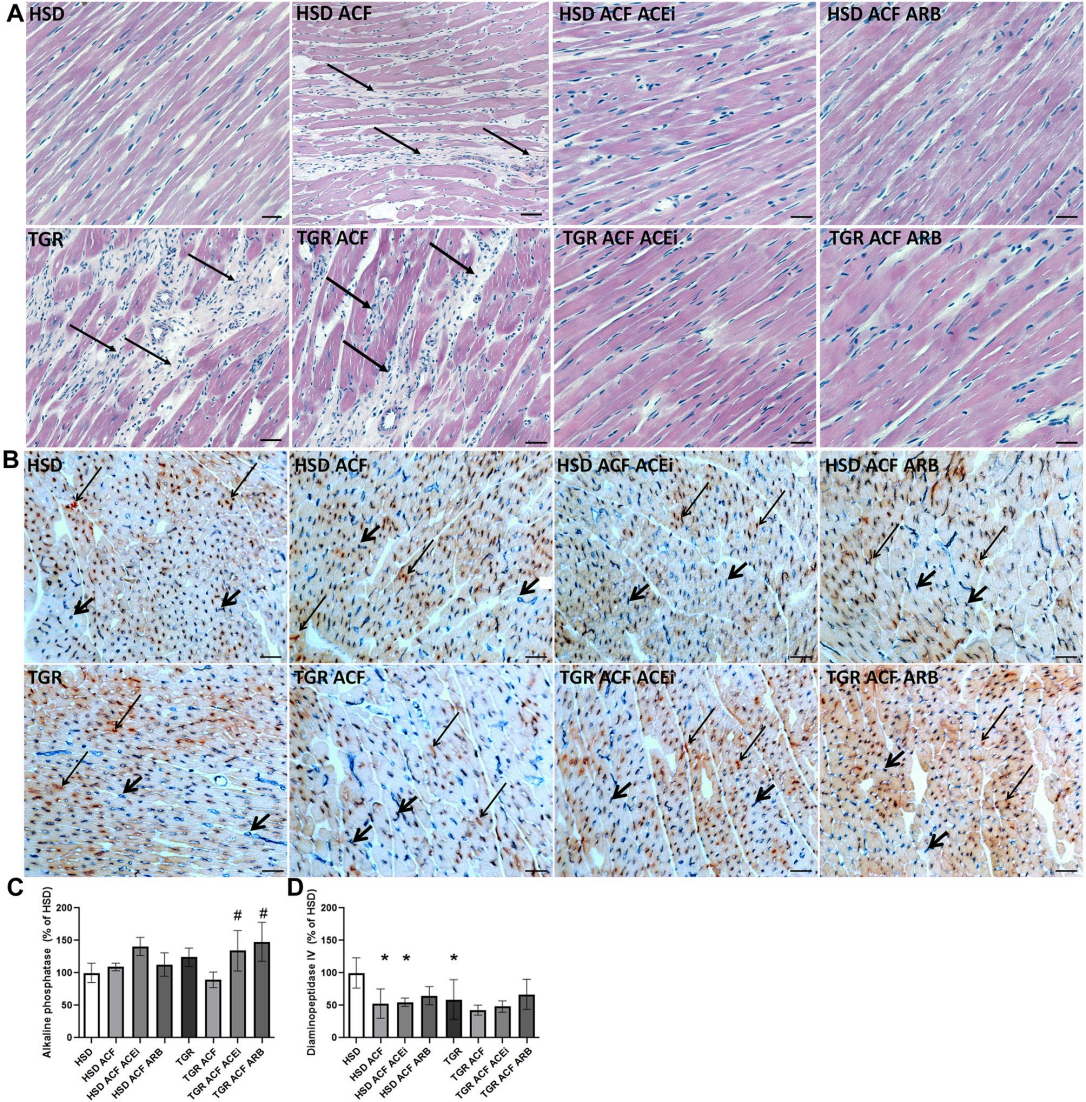
(**A**) Hematoxylin–eosin staining, and dynamic alterations in the ECM network (arrows). (**B**) Histochemical demonstration of dipeptidyl peptidase-4 (DPPIV, brown, longer arrows) and the alkaline phosphatase activity (AP, blue, shorter arrows) in endothelial cells of the venous (DPPIV) and arterial portion (AP) of capillaries and quantification (**C,D**) of the reaction’s intensity. *HSD* normotensive Hannover Sprague–Dawley rats, *TGR* hypertensive heterozygous Ren-2 transgenic (mREN2)27 rats, *ACF* aortocaval fistula, *ECM* extracellular matrix, *ACEi* angiotensin-converting enzyme inhibitors, *ARB* angiotensin II Receptor Blockers. Scale bar represents 200 µm. 10 µm thick frozen tissue sections from the apex of the heart were used. Results are the mean ± SD (sample size is illustrated in Supplementary Fig. 1), *p < 0.05 vs. HSD/TGR, ^#^p < 0.05 vs. ACF. TGR ACF rats suffered with higher incidence of mortality (60%).

Representative microscopic images of hematoxylin–eosin stained left ventricular tissue of experimental rats are shown in Fig. 1A. There is prevalent population of hypertrophied cardiomyocytes and occurrence of dynamic alterations in the ECM network in TGR versus HSD rats. Moreover, alterations in the ECM network are abundant in ventricular tissue due to ACF-induced HF in HSD rats and even more pronounced in TGR. Treatment with trandolapril and losartan partially attenuated ECM changes in both rat strains.

In situ histochemical demonstration of alkaline phosphatase (AP) activity, which points out the function of the arterial part of capillaries as well as the activity of dipeptidyl peptidase IV (DPPIV), reflecting function of venous part of capillary network, is demonstrated in Fig. 1B. Activity of AP (blue colour) was reduced in response to ACF-induced HF only in TGR heart but increased due to treatment with losartan and trandolapril in both rat strains (Fig. 1C). QIA revealed decreased activity of DPPIV (brown colour) in the left ventricle of TGR versus HSD rats as well as in both rat strains following ACF-induced HF. It was not affected by treatment with either drug (Fig. 1D).

**Figure 2:**
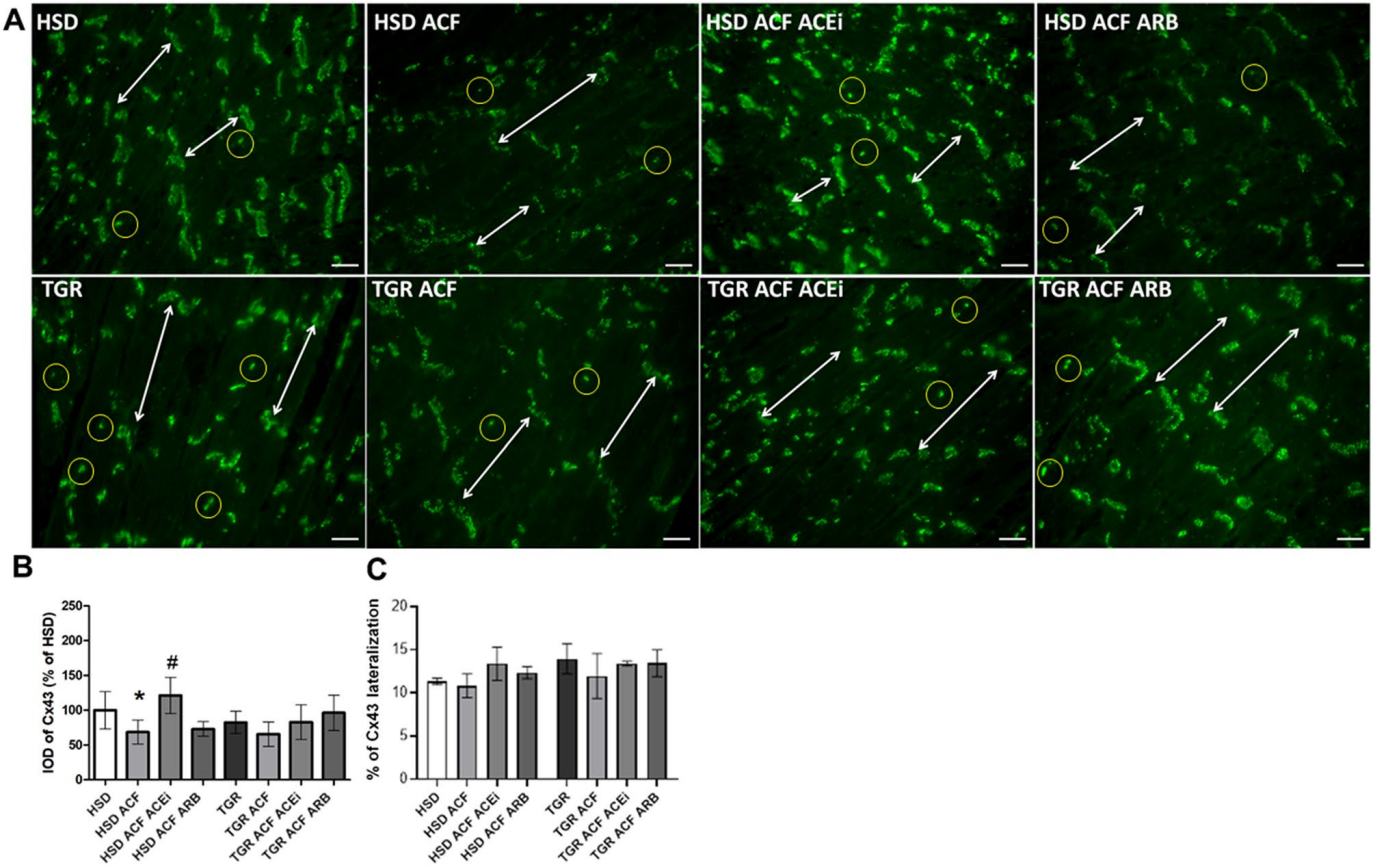
(**A**) Visualisation of the myocardial connexin-43 (Cx43, green) using immunofluorescence staining. Double arrows represent Cx43 (green) at the intercalated discs of the cardiomyocytes. Some lateral topology of Cx43 is highlighted by yellow circles. (**B**) Graph represents a total integral optical density per area (IOD) of Cx43. (**C**) Graph represents a percentage of lateralized connexin-43 in myocardial tissue. *HSD* normotensive Hannover Sprague–Dawley rats, *TGR* hypertensive heterozygous Ren-2 transgenic (mREN2)27 rats, *ACF* aortocaval fistula, *ACEi* angiotensin-converting enzyme inhibitors, *ARB* angiotensin II Receptor Blockers. Scale bar represents 200 µm. 10 µm thick frozen tissue sections from the apex of the heart were used. Results are the mean ± SD (sample size is illustrated in Supplementary Fig. 1), *p < 0.05 vs. HSD/TGR, ^#^p < 0.05 vs. ACF. TGR ACF rats suffered with higher incidence of mortality (60%).

Representative microscopic images of Cx43 immunolabeling are demonstrated in Fig. 2A. There is a prevalent Cx43 localisation at the intercalated discs and sporadic on lateral sides of the cardiomyocytes that is obvious in healthy heart. Unlike hypertensive TGR, in which lateral localisation of Cx43 was enhanced in hypertrophied cardiomyocytes (Fig. 2A). According to QIA, lateral topology of Cx43 did not change significantly in response to ACF-induced HF regardless the rat strain (Fig. 2A,C). While Cx43 immunofluorescence signal was decreased in response to ACF-induced HF in HSD rat heart only (Fig. 2A,B). Moreover, there was a tendency to increase lateralization of Cx43 after treatment with either drug (Fig. 2A,C).

### Myocardial levels of proteins implicated in modulation of intercellular communication and extracellular matrix

**Figure 3:**
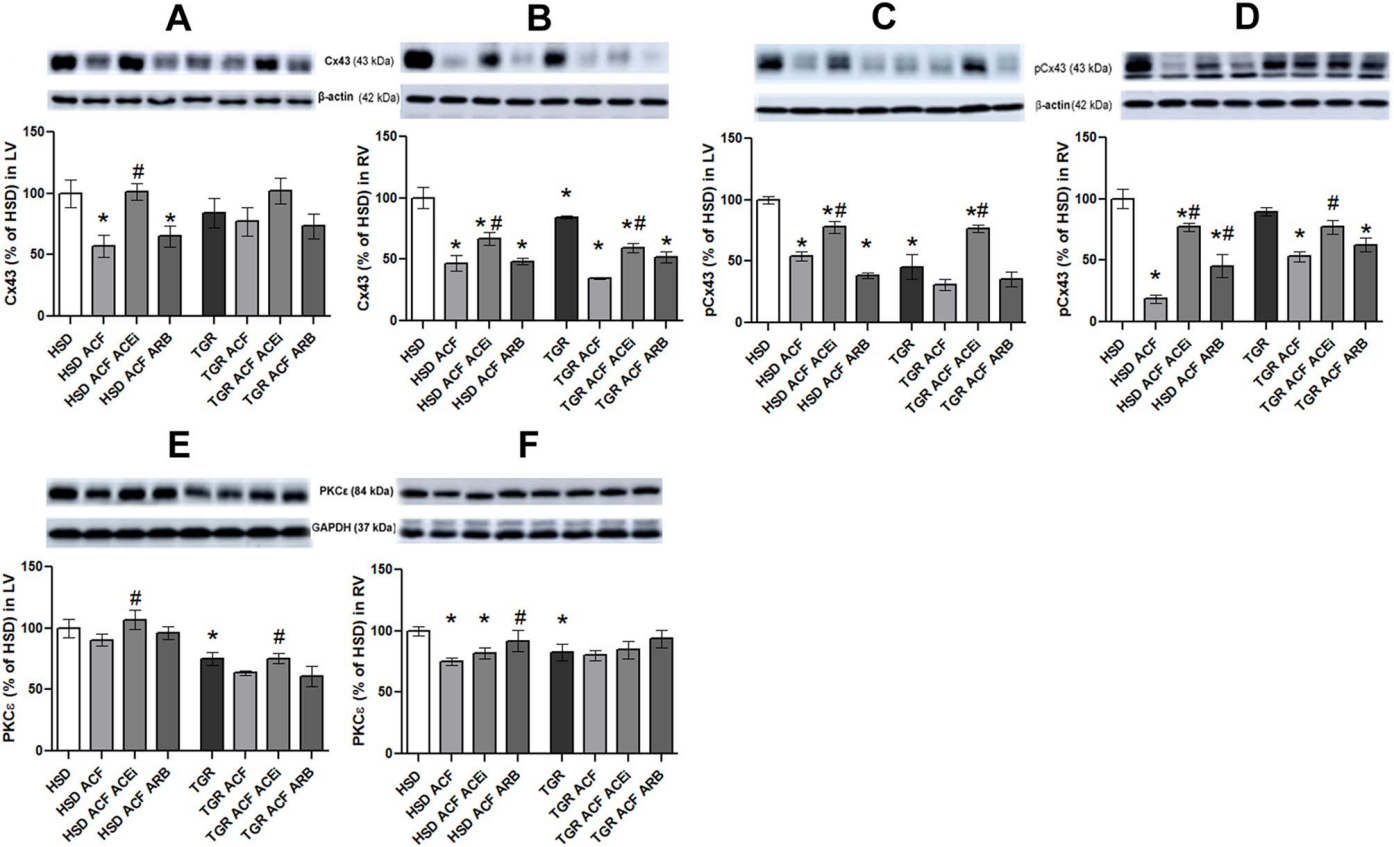
Western blot demonstration of total Cx43 (**A,B**), its functional phosphorylated forms (**C,D**) and PKCε (**E,F**) protein levels in the experimental rats following ACF. *HSD* normotensive Hannover Sprague–Dawley rats, *TGR* hypertensive heterozygous Ren-2 transgenic (mREN2)27 rats, *ACF* aortocaval fistula, *ACEi* angiotensin-converting enzyme inhibitors, *ARB* angiotensin II Receptor Blocker, *Cx43* connexin-43, *PKCε* protein kinases C epsilon, *LV* left ventricular tissue, *RV* right ventricular tissue. Results are the mean ± SD (sample size is illustrated in in Supplementary Fig. 1), *p < 0.05 vs. HSD/TGR, ^#^p < 0.05 vs. ACF. TGR ACF rats suffered with higher incidence of mortality (60%).

Protein abundance of total Cx43 and its variant phosphorylated at serine-368 were decreased in right as well as left ventricles of TGR versus HSD rats (Fig. 3A–D). Moreover, comparing to sham rats, ACF-induced HF reduced these myocardial proteins in both rat strains. In parallel, protein abundance of PKCε, (one of the protein kinases that can phosphorylate Cx43 at serine-368) was reduced in TGR versus HSD rat hearts (Fig. 3E,F). ACF-induced HF did not affect significantly PKCε protein levels in TGR, while reduced it in right ventricle of HSD rat heart. Treatment with trandolapril significantly attenuated alterations of Cx43, its P-ser368 variant as well as PKCε in both rat strains suffering from HF, while losartan did not affect examined myocardial proteins significantly regardless the rat strain.

**Figure 4:**
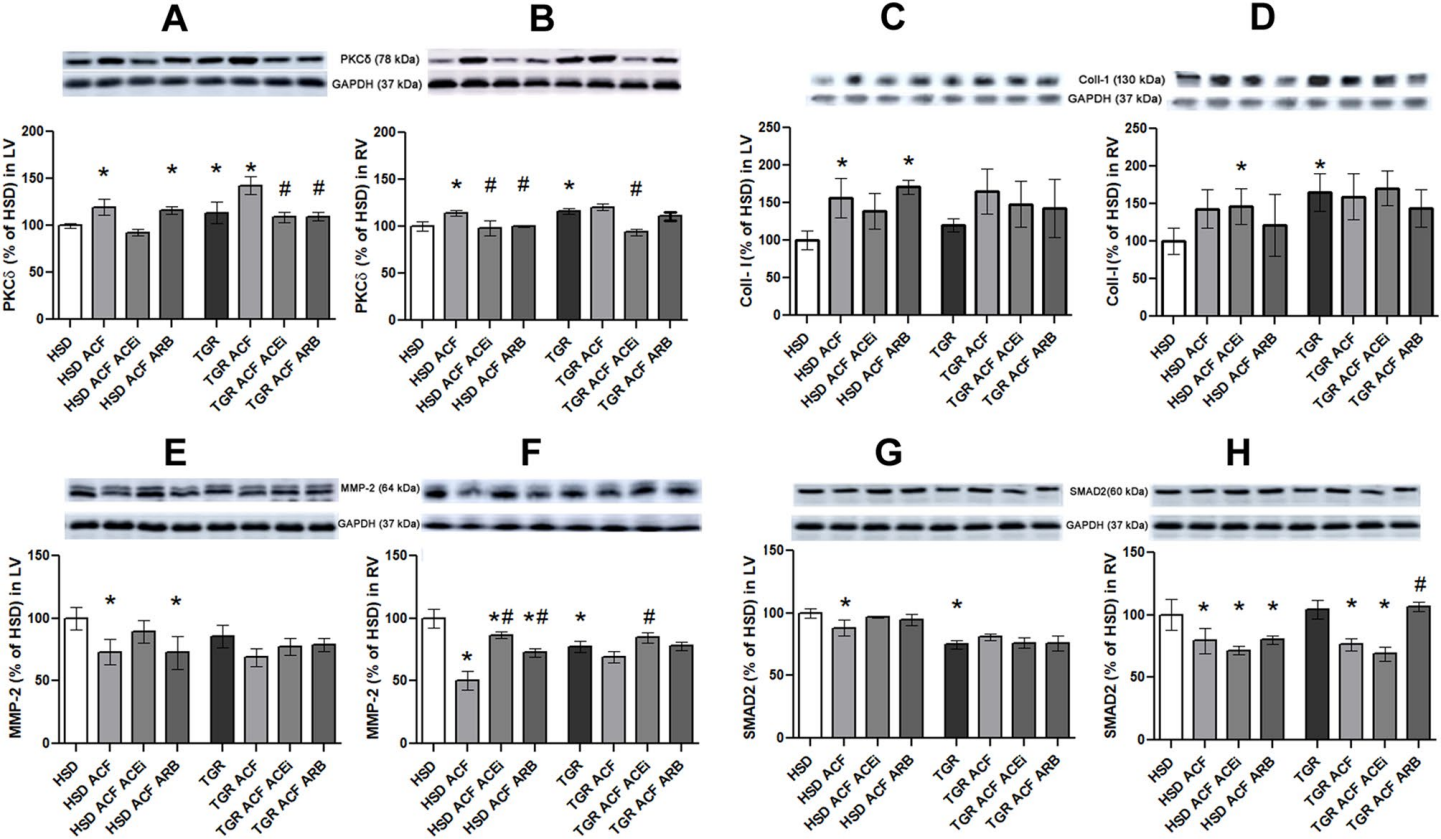
Western blot demonstration of PKC delta (**A,B**), collagen-1 (**C,D**), MMP-2 (**E,F**), SMAD 2 (**G,H**) protein levels in the experimental rats following ACF. *HSD* normotensive Hannover Sprague–Dawley rats, *TGR* hypertensive heterozygous Ren-2 transgenic (mREN2)27 rats, *ACF* aortocaval fistula, *ACEi* angiotensin-converting enzyme inhibitors, *ARB* Angiotensin II Receptor Blocker, *PKC delta* protein kinases C delta, *Coll-1* collagen 1, *MMP-2* matrix metalloproteinase 2, *SMAD 2* Signal transducer for receptors of the transforming growth factor beta, *LV* left ventricular tissue, *RV* right ventricular tissue. Results are the mean ± SD (sample size is illustrated in Supplementary Fig. 1), *p < 0.05 vs. HSD/TGR, ^#^p < 0.05 vs. ACF. TGR ACF rats suffered with higher incidence of mortality (60%).

Collagen-1 as well as pro-fibrotic PKCδ protein levels were higher in TGR versus HSD rat hearts (Fig. 4A–D). ACF-induced HF increased abundance of myocardial collagen-1 and PKCδ in both rat strains but not in right ventricle of TGR. Moreover, there was an increase of hydroxyproline content in response to ACF-induced HF in both rat strains. Treatment with either trandolapril or losartan reduced hydroxyproline significantly in right and to a lesser extent in left ventricle of HSD as well as TGR heart (Table 1). However, treatment with either drug did not affect elevated collagen-1 in both rat strains (Fig. 4C,D), while reduced protein levels of PKC δ in response to ACF-induced HF (Fig. 4A,B).

Protein abundance of SMAD2/3 and MMP-2 was lower in TGR compared to HSD rat hearts (Fig. 4E–H). The SMAD2/3 and MMP-2 protein levels were also decreased in RV and LV heart tissue of control and hypertensive rats with detected volume overload heart failure. Treatment did not significantly affect both pro-fibrotic factors in the LV of TGR and ACF-affected HSD and TGR rat hearts but the significance was reached in the RV heart tissue of ACF-affected rats (Fig. 4A–H). However, circulating levels of MMP-2, and its inhibitor TIMP-2 were not changed in TGR and ACF-affected HSD and TGR rats, while slightly by ACEi (Table 1).

## Discussion

Findings of the current study refer to high-output congestive HF that was established due to ACF-induced biventricular volume overload in normotensive HSD and hypertensive TGR. Indeed, echocardiography revealed decline in heart function, in both rat strains, according to significantly reduced ejection fraction, while cardiac output was increased, comparing to sham rats^25^. It is in line with data reported in Sprague–Dawley rats^27^. HF was accompanied by an increase of natriuretic and diuretic hormones, BNP-45 and ANP causing vasorelaxation. Secretion of ANP and BNP-45 is a direct response to wall stretch. BNP-45 is a more sensitive marker to hemodynamic stress. The increase of the BNP-45 occurs acutely within 1 h. For this reason, changes in BNP-45 compared to ANP can be much more pronounced in our model of HF^28^.

Moreover, serum and heart tissue lipid peroxidation marker, TBARS, was elevated in the rats with ACF-induced HF, regardless the rat strain. In accordance with ACF-induced increase of mitochondrial ROS production^29,30^. ACF induced increase in lungs, right and left ventricle mass index, in HSD as well as TGR, shown previously in normotensive rats^10,15,27,31,32^. These parameters were utilized as markers of hypertrophy^33^ and suggesting a consistent development of HF^34^. Besides, TGR rats with ACF-induced HF exhibited significant reduction in body weight most likely associated with higher mortality incidence. Cardiac cachexia is a serious complication of HF associated with an impaired survival rate^35^.

There was a clear benefit of treatment either with ACEi (trandolapril) or ARB (losartan), which significantly improved ejection fraction in ACF-affected TGR and HSD rats^25^. Treatment with either drug supressed circulating BNP-45, ANP and TBARS levels in both heart ventricles and reduced lung and heart mass index, including right and left ventricles regardless the rat strain. Likewise in HSD rats treated with trandolapril^31^. Besides that, treatment increased alkaline phosphatase activity in HSD and TGR rats, responsible for the breakdown of phosphate esters and transfer of phosphate (e.g. adenosine through cell membrane. Adenosine acts as a vasodilator and antiplatelet agent). It has been shown that the activity of alkaline phosphatase was reduced in the myocardium of hypertensive rats with cardiac hypertrophy and fibrosis^36,37^. Generally, in all forms of heart failure, including hypertrophic and dilated cardiomyopathy, coronary blood flow impairment is present^38^. It appears that treatment contributes to the improvement of heart function via an enhanced capillary network to deliver substrate and oxygen^37,39^.

ACF induced decline of Cx43 protein levels in HSD rat heart ventricles, consistent with previous findings^10,15,27^. Moreover, there was a decrease of Cx43 variant phosphorylated at serine368 in both heart ventricles. Unlike to HSD rats, the decline of Cx43 protein and its serine368 variant was significant in right, but not in left ventricle of TGR suggesting strain-and heart chamber-related difference in response to ACF. Nevertheless, treatment particularly with trandolapril significantly attenuated down-regulation of Cx43 protein and its ser-368 variant in HF suffering rats of both strains. Besides that, treatment with trandolapril mitigated down-regulation of PKCε. This protein kinase is one of the kinases implicated in the phosphorylation of Cx43, however, the detection of PKCε activity to show a direct association between Cx43 ser-368 and PKCε is missing.

Noteworthy, Cx43 ser-368 variant was associated with anti-arrhythmic phenotype^17,18,40^ most likely due to maintenance of Cx43 channels mediated electrical coupling at the intercalated discs.

It has been established that down-regulation of Cx43 as well as its abnormal topology contribute to the arrhythmogenic substrate in failing human heart promoting occurrence of life-threatening arrhythmias^9,41^. Enhanced distribution of Cx43 on lateral sides of the hypertrophied cardiomyocytes of along with reduced Cx43 protein levels may hamper conduction^42^ and likely to be associated with prolongation of QRS interval in response to ACF^12,31^, thereby increase a risk to develop potentially lethal VF^43^. In this context it should be noted that we did not observe enhanced “lateralization” of Cx43 in hypertrophied cardiomyocytes of ACF-affected HSD rats. While such abnormal distribution of Cx43 was induced by pressure overload in TGR, likewise in other spontaneously hypertensive rat strain^17,18,39^. It suggests difference in Cx43 topology (lateralization) between volume overload induced eccentric versus pressure overload induced concentric hypertrophy. Of interest, myocardial expression of Cx43 was reduced in patients with dilated cardiomyopathy prone to sudden cardiac death^44^. Considering Cx43 as a key factor impacting susceptibility of the heart to life-threatening arrhythmias^13^, our findings challenge to explore susceptibility of the heart to the formation of arrhythmogenic substrate in both ACF affected rat strains as well as to examine pleiotropic anti-arrhythmic potential of trandolapril and losartan. These drugs, in addition, attenuated elevation of the pro-hypertrophic PKCδ protein levels in response to ACF.

In regard to the vulnerability of the failing heart to malignant arrhythmias another crucial pro-arrhythmic factor should be considered, i.e. remodelling of extracellular matrix that hampers Cx43 mediated intermyocyte electrical coupling and also deteriorates heart function^45,46^. In this context it should be noted that a marker of myofibroblasts, periostin, as well as transglutaminase involved in crosslinking of ECM proteins, were upregulated in both heart ventricles of ACF-affected normotensive rats^32^. In contrast, hypofibrotic cardiac fibroblast phenotype due to volume overload was reported by others^47^. Of interest, myocardial SMAD2/3 proteins implicated in pro-fibrotic signalling were decreased in TGR versus HSD. It is in line with reported decreased nuclear translocation of transcriptional regulators^48^.

Collagen-1 increased in response to ACF in HSD rat hearts only, while its elevation in TGR was not affected by ACF. Altogether, it points out on strain related difference in the levels of ECM markers and in response to volume overload. The content of collagen associated hydroxyproline that is substrate of MMPs, was increased but MMP2 protein decreased in both rat strains in response to ACF, suggesting of ECM remodelling. The ECM alterations were attenuated by treatment with trandolapril or losartan. Nevertheless, the extent of ECM remodelling and its impact on heart function and arrhythmogenesis in condition of pressure and volume overload should be more thoroughly investigated. Taken into account an important interaction between degradation of interstitial collagen in acute volume overload and disorders of subsarcolemmal mitochondria function could contribute to progression of HF^30^.

In conclusion, main findings of this study clearly point out the differences of normotensive versus hypertensive rat hearts in response to volume overload, while the former are more responsive. Apparent down-regulation of myocardial Cx43 along with ECM remodelling may contribute not only to HF but also to the formation of an arrhythmogenic substrate. Noteworthy, both trandolapril and losartan significantly attenuated pressure and volume overload-induced Cx43 and ECM alterations that may contribute to improvement of heart function. Moreover, it also indicates pleiotropic anti-arrhythmic potential of both currently used cardioprotective drugs that requires further attention. Of note, trandolapril exhibited a more pronounced effect which can be attributed to significant suppression of Angiotensin II plasma levels and c-Src kinase that is involved in Cx43 down-regulation and hampering mutual cardiomyocyte communication, thereby providing a substrate for arrhythmia^49^. Malignant arrhythmias and HF are growing and coexisting pathologies that require permanent attention of both clinical and experimental cardiologists. We showed, for the first time, the impact of simultaneous, volume and pressure overload on examined factors, in normotensive and hypertensive rats, as well as cardioprotective effects of treatments with trandolapril and losartan.

## Limitation of the study

The main limitation of this study is missing examination of electrocardiographic parameters and testing of cardiac arrhythmia susceptibility that require additional set of rats. It will be our priority in our future study.

## Materials and methods

### Experimental animals, ACF-induced HF and design of the experiment

Rats were bred, maintained, and handled at the Centre of Experimental Medicine of Institute of Clinical and Experimental Medicine (IKEM), Prague and approved on 26 June 2017 by the Animal Care and Use Committee of the IKEM, Prague, project number 50/2017, in accordance with guidelines and practices established by the Directive 2010/63/EU of the European Parliament on the Protection of Animals Used for Scientific Purposes. This study was conducted in compliance with the ARRIVE 2.0 Guidelines for Reporting Animal Research^50,51^.

Rats were housed under standard conditions, at 22 ± 1 °C, 12-h light/dark cycles, with ad libitum access of rat chows and tap water. In the experiment, 80 males of the hypertensive heterozygous Ren-2 transgenic (mREN2)27 rats (TGR, n = 40) and normotensive Hannover Sprague–Dawley rats (HSD, n = 40) were used.

As previously reported^25^**,** HF due to volume overload was induced by creation of an aortocaval fistula (ACF) between the abdominal aorta and inferior vena cava^52^. Surgical procedures were performed under general anaesthesia by an intraperitoneal administration of ketamine (Calypsol, Gedeon Richter, Hungary, 160 mg/kg) and midazolam (Dormicum, Roche, France, 160 mg/kg). In the 8 weeks old HSD and TGR rats, shunt was created by needle technique between vessels (18-gauge needle, 1.2 mm in diameter) and subsequently sealed with cyanoacrylate glue (Histoacryl, B.Braun AG, Germany). ACF induction was confirmed by recording of pulsatile flow in the vena cava. Control HSD and TGR rats underwent a sham operation. After five weeks from successful ACF induction, while heart failure was fully developed in rats, 15 weeks of treatment has begun. HSD and TGR rats with ACF were treated with an AT1 receptor blocker (200 mg/l, losartan, Lozap, Zentiva, Prague, CZ) or an ACE inhibitor trandolapril (6 mg/l, Gopten, Mylan, Canonsburg, Pennsylvania, USA), drugs dissolved in drinking water. Age matched controls without treatment were used. Rats were decapitated at the age of 28 weeks. At the end of the experiment, whole heart weight, left ventricle weight, right ventricle weight and lung weight (“wet lung weight”) were recorded. Blood and heart tissue were collected for further analyses (Supplementary Fig. 1).

### Assessment of circulating levels of atrial natriuretic peptide (ANP)

ANP as a marker of HF, mainly synthesized in the atria, was estimated in blood serum samples according to the manufacturer’s recommended protocol (Atrial Natriuretic Peptide EIA Kit, RAB00385, Sigma-Aldrich, St. Louis, USA). Briefly, 100 μl of anti-ANP antibody was pipetted into the included secondary antibody-coated microplate. After incubation and anti-ANP antibody removal, 100 μl of blood serum/standards was added. After these steps followed incubations with 100 μl of HRP-streptavidin solution, and 100 μl of TMB solution (3,3,5,5-tetramethylbenzidine). Finally, 50 μl of STOP solution was added and the absorbance was measured with a spectrophotometer (Synergy H1, BioTek, USA) at 450 nm. The amount of ANP (pg/ml) in the samples was calculated using a regression equation from the values of the standards.

### Assessment of circulating levels of brain natriuretic peptide 45 (BNP-45)

BNP-45 as a marker of HF, mainly released from the ventricles, was estimated in blood serum samples according to the manufacturer’s recommended protocol (Rat BNP 45 ELISA Kit, Canbrige, UK). Briefly, 50 µl of the standards and blood serum samples into the wells of the microtitre plate and let them incubate for 120 min at room temperature. After incubation, we washed the wells five times with washing solution and then added 50 µl of biotinylated BNP-45 antibody to all the wells that we analyzed for 120 min. Subsequently, we washed the plate again five times with washing solution and added 50 µl of HRP-Streptavidin solution for 30 min. We washed the plate five times with washing solution and added 50 µl of chromogenic substrate for 8 min, and terminated the reaction by adding 50 µl of STOP solution. Absorbance was measured with a spectrophotometer (Synergy H1, BioTek, USA) at a wavelength of 450 nm. The amount of BNP (pg/ml) in the samples was calculated using the regression equation from the values of the standards.

### Assessment of circulating levels of tissue inhibitor of metalloproteinase 2 (TIMP-2)

We analyzed TIMP-2 using the commercial Rat TIMP-2 ELISA Kit for cell culture supernatants, plasma, and serum samples (RAB1156, Sigma-Aldrich, St. Louis, USA) according to the recommended procedure. We added 100 µl of the standards and blood serum samples to the wells of the microtitre plate, and let them incubate for 150 min at room temperature with gentle mixing. After incubation, the contents of the wells were removed and washed four times with washing solution. Subsequently, we added 100 µl of biotinylated antibody to the wells and incubated for an hour at room temperature. After incubation, the content of the wells was removed and washed four times with washing solution. We immediately added 100 µl of the HRP-Streptavidin solution and let it incubate for 45 min at room temperature with gentle mixing. Even after this step, the contents of the wells were removed and washed four times with washing solution. Immediately after washing, we added 100 µl of TMB solution to the wells and let them incubate for 30 min at room temperature with gentle mixing. Finally, we added 50 µl of STOP solution to the wells of the microtitre plate and measured the absorbance with a spectrophotometer (Synergy H1, BioTek, USA) at a wavelength of 450 nm. The amount of TIMP-2 (pg/ml) in the samples was calculated using the regression equation from the values of the standards.

### Determination of thiobarbituric acid reactive substances (TBARS)

TBARS levels, as a marker for lipid peroxidation, were analysed as was described previously Shlafer and Shepard (1984) with modifications by spectrophotometric method. Briefly, 40 µl of standards, blood serum samples or tissue homogenates (same as in SDS-PAGE method) from RV and LV together with 40 µl of 20% trichloroacetic acid solution were mixed with a quadruple volume of TBARS reagent (37 mmol/l C_4_H_4_N_2_O_2_S; 500 mmol/l NaOH; 15% v/v CH_3_COOH) and incubated for 70 min at 100 °C. After cooling the samples for 20 min, samples were pipetted into another tube with prepared mixture of n-butanol and pyridine (14:1, v/v), and subsequently centrifuged at 5000×*g* for 10 min. The resulting supernatant (organic phase) was used for measurement of absorbance at 535 nm by Synergy H1 Hybrid Multi-Mode Microplate Reader (Biotek, Vermont, USA). The concentration of malondialdehyde (MDA) in the samples was calculated from the calibration curve formed from a tetrabutylammonium malondialdehyde salt^53,54^.

### SDS-PAGE and western blotting

As was described in details in our previous works^16^, approximately 100 mg of frozen heart tissue (RV, LV) was homogenized in lysis buffer (20% SDS, 10 mmol/l EDTA, 100 mmol/l Tris, pH 6.8) and diluted in Laemmli sample buffer under reducing and non-reducing conditions. Protease inhibitor cocktail was used as well (Sigma-Aldrich, St.Louis, MO, USA, #P8340). Loading equal amounts of protein (6–60 µg) per lane were separated in 10% SDS–polyacrylamide gels at a constant voltage of 90 V (Mini-Protean TetraCell, Bio-Rad, Hercules, CA, USA) and electrically transferred to a nitrocellulose membrane (0.2 m pore size, Advantec, Tokyo, Japan). Membranes were blocked with 5% low-fat milk and incubated with the appropriate primary and secondary antibodies (Table 1). Proteins visualization was carried out by enhanced chemiluminescence method and quantification by densitometric analysis using Carestream Molecular Imaging Software (version 5.0, Carestream Health, New Haven, CT, USA).

### MMP-2 activity by gelatin zymography

The gelatinolytic activity of MMP-2 was analyzed by zymography in polyacrylamide gels with gelatin as a substrate. Laemmli buffer without 2-mercaptoethanol was added to the protein samples, and non-heated samples were subjected to electrophoresis in SDS–polyacrylamide gels with gelatin (2 mg/ml). After electrophoresis, the gels were washed twice for 20 min each with 50 mmol/l Tris–HCl (pH 7.4), containing 2.5% Triton X-100, and then incubated overnight at 37 °C in a substrate buffer containing 50 mmol/l Tris–HCl, 10 mmol/l CaCl2 and 1.25% Triton X-100, pH 7.4. After incubation, the gels were stained with 1% Coomassie Brilliant Blue G-250 and then destained with 40% methanol and 10% acetic acid. The gelatinolytic activity of the MMP-2 was detected as transparent bands against a dark blue background^55^.

### Immunofluorescence methods and quantitative image analysis (QIA)

As was described in our previous works, for immunodetection of Cx43, we used a 10 µm thick frozen tissue sections from the apex of the heart. Cryosections were fixed in ice-cold methanol, permeabilized in 0.3% Triton X-100, and blocked with the solution of 1% bovine serum albumin. Tissue sections were incubated with primary antibody and then with secondary antibody (Table 1). For visualization of actin filaments, phalloidin (Sigma-Aldrich, St.Louis, MO, USA, #P2141) was applied to the sections. At the end of procedure were tissue sections mounted in the Vectashield medium (H-1200, Vector Laboratories-Inc., Burlingame, CA, USA) and detected by Zeiss Apotome 2 microscope (Carl Zeiss, Jena, Germany).

For QIA ten randomly acquired images per heart were examined. Immunofluorescence signal was defined as a number of pixels with the protein signal intensity exceeding a threshold of 30 on the 0–255 Gy scale. The total number of positive pixels was indicated as a total integral optical density per area (IOD).

Quantification of Cx43 lateralization was manually performed by selection and delineation of terminal intercalated disc-related areas of Cx43. IOD of lateral Cx43 immunofluorescence signal (Image-Pro Plus) corresponds to the difference between total IOD per area and IOD of terminal areas. Lateralization of Cx43 was calculated from the ratio of IOD of lateral topology divided by the total IOD and expressed in percentage^39,56^.

For better visualization of Cx43 topology, not used for QIA, we replaced in the representative images, the green colour of Cx43 located at the intercalated discs of the cardiomyocytes to white colour and laterally oriented Cx43 are visible in green colour. Some selected lateral topology of Cx43 is highlighted by yellow circles (Supplementary Fig. 2).

### Determination of collagen content by hydroxyproline measurement

The hydroxyproline content considered as a marker of fibrosis was measured by a spectrophotometric method as described previously. Approximately 100 mg of myocardial tissue from LV and RV was dried overnight and hydrolysed in 6 M HCl. Samples were dried again and incubated with 4 M NaOH, Chloramine T in the acetate-citrate buffer. After oxidation reaction were samples incubated with Ehrlich’s reagent solution. Samples were pipetted onto a microplate and final concentration of hydroxyproline was measured spectrophotometrically at 550 nm. The hydroxyproline content was then expressed in mg per total weight of the LV or RV^17,53^.

### Histology and enzyme histochemistry of myocardial tissue

10 µm thick tissue cryosections from the apex were used for conventional hematoxylin–eosin staining and catalytic enzyme histochemistry performed according to Lojda (1979) with modifications. For hematoxylin–eosin reaction to viewing tissue structure were tissues fixed in 4% buffered formaldehyde, stained with hematoxylin–eosin solutions, poured with gelatin and covered with a coverslip.

Endothelium-related alkaline phosphatase (AP, E.C.3.1.3.1) with naphthol AS-MX phosphate as a substrate and dipeptidyl peptidase-4 (DPP4, E.C.3.4.15.4) with glycyl-l-proline-4-methoxy-beta naphthylamide as a substrate were used to detect activities arteriolar and venular capillary network. Cryosections were incubated in solution (1.2 mM l-Leucine 4-methoxy-β-naptylamide hydrochloride; 5% dimethylformamide; 2.4 mM Fast blue BB; 0.1 M Na_2_ HPO_4_ × 2H_2_O; 1 M KH_2_PO_4_), poured with gelatin and covered with a coverslip.

Examination of stained sections were observed under light microscope (Zeiss Apotome 2 microscope Carl Zeiss, Jena, Germany). Ten randomly acquired microscopic images per heart were used for quantification^39,57^.

### Statistical analysis

All data are expressed as mean ± SD. Kolmogorov–Smirnov normality test to examine if variables are normally distributed was used. Differences between groups were detected by one-way ANOVA, followed by Bonferroni’s posthoc test. p < 0.05 was considered statistically significant.

### Institutional review board statement

All animal experiments were approved on 26 June 2017 by the Animal Care and Use Committee of the Institute for Clinical and Experimental Medicine, Prague; project number 50/2017; in accordance with guidelines and practices established by the Directive 2010/63/EU of the European Parliament on the Protection of Animals Used for Scientific Purposes.

### Supplementary Information

Supplementary Information 1.

Supplementary Information 2.

## Data Availability

The datasets generated during and/or analyzed during the current study are available from the corresponding author on reasonable request.
