# Supplementary material for: The treatment with trandolapril and losartan attenuates pressure and volume overload alternations of cardiac connexin-43 and extracellular matrix in Ren-2 transgenic rats
Source: Sci Rep. 2023 Nov 27;13:20923. doi: 10.1038/s41598-023-48259-2 (PMC10684879; doi:10.1038/s41598-023-48259-2)
Supplement: Supplementary file 1 — Supplementary Information 1. [file 41598_2023_48259_MOESM1_ESM.pdf]

GAPDH

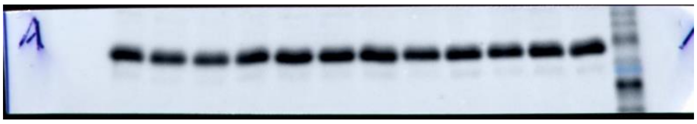

HSD HSD ACF HSD ACF ACEi HSD ACF ARB

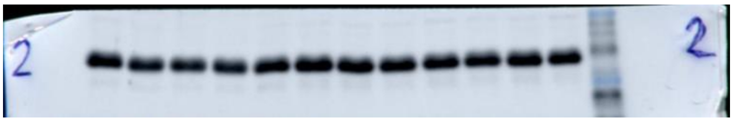

HSD TGR TGR ACF TGR ACF ACEi TGR ACF ARB

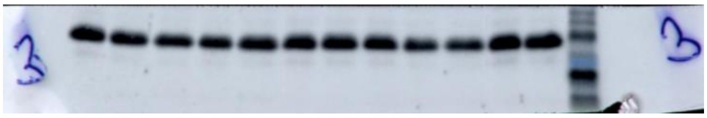

HSD HSD ACF HSD ACF ACEi HSD ACF ARB

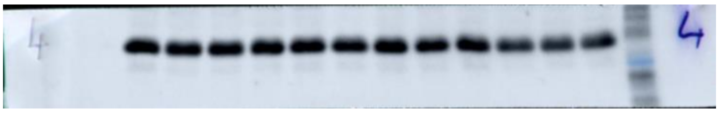

HSD TGR TGR ACF TGR ACF ACEi TGR ACF ARB

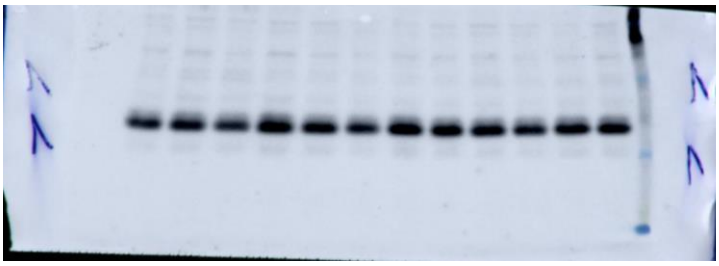

HSD HSD ACF HSD ACF ARB HSD ACF ACEi

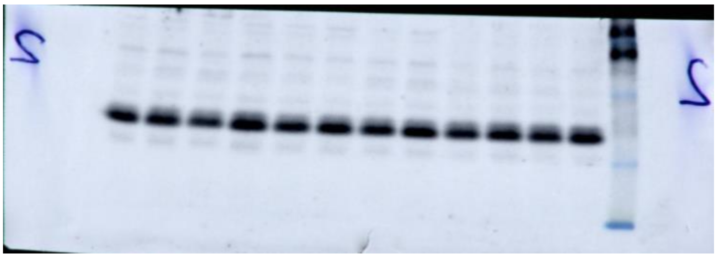

HSD TGR TGR ACF TGR ACF ARB TGR ACF ACEi

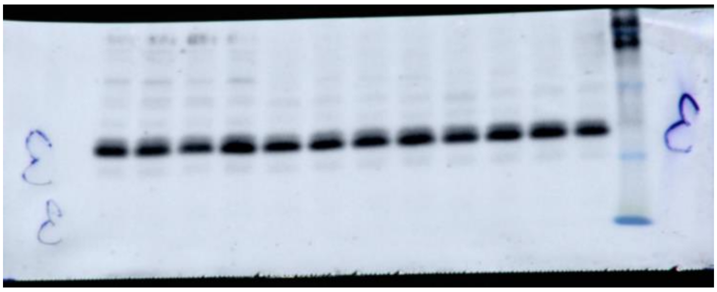

HSD HSD ACF HSD ACF ARB HSD ACF ACEi

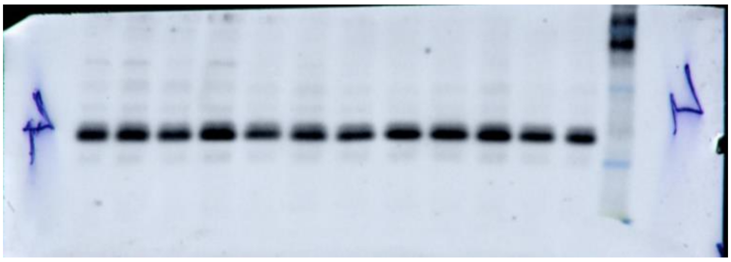

HSD TGR TGR ACF TGR ACF ARB TGR ACF ACEi

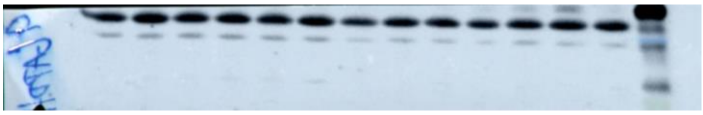

HSD HSD ACF HSD ACF ACEi HSD ACF ARB

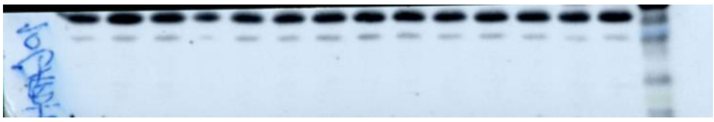

TGR HSD TGR ACF TGR ACF ACEi TGR ACF ARB

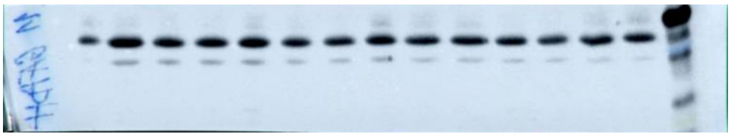

HSD HSD ACF HSD ACF ACEi HSD ACF ARB

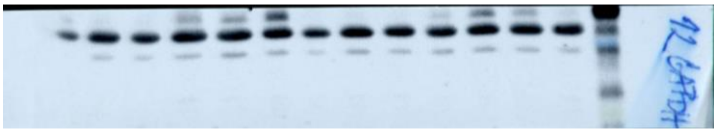

TGR HSD TGR ACF TGR ACF ACEi TGR ACF ARB

MMP-2 - LV

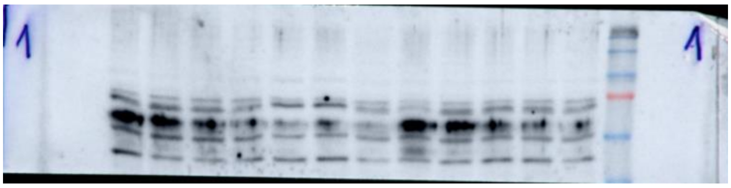

HSD HSD ACF HSD ACF ACEi HSD ACF ARB

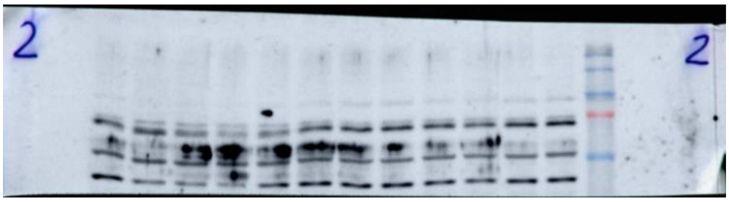

HSD TGR TGR ACF TGR ACF ACEi TGR ACF ARB

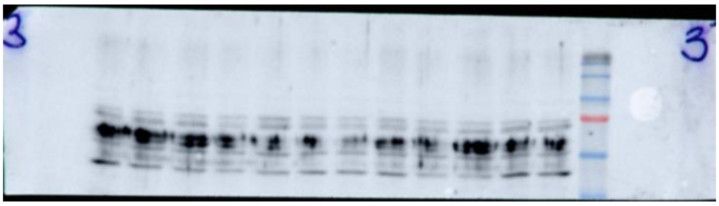

HSD HSD ACF HSD ACF ACEi HSD ACF ARB

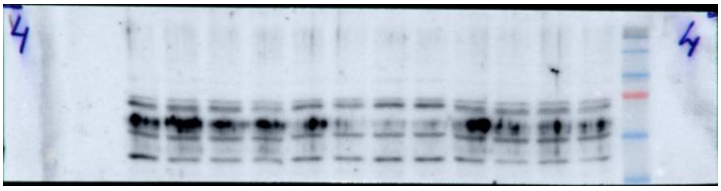

HSD TGR TGR ACF TGR ACF ACEi TGR ACF ARB

MMP-2 - RV

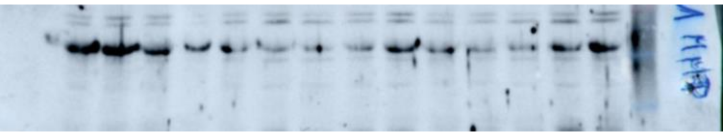

HSD HSD ACF HSD ACF ACEi HSD ACF ARB

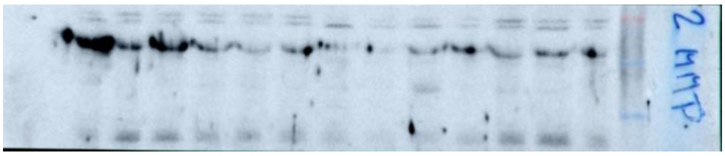

HSD TGR TGR ACF TGR ACF ACEi TGR ACF ARB

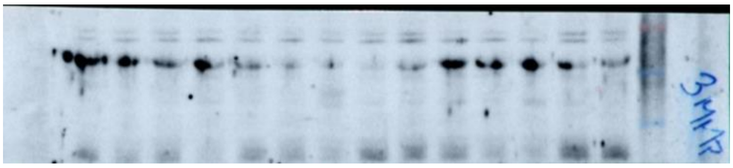

HSD HSD ACF HSD ACF ACEi HSD ACF ARB

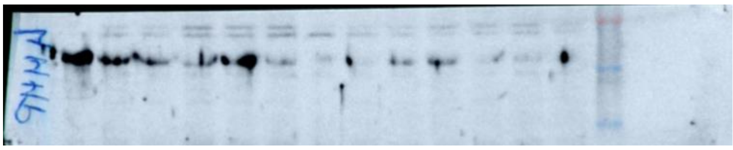

HSD TGR TGR ACF TGR ACF ACEi TGR ACF ARB

PKCe - LV

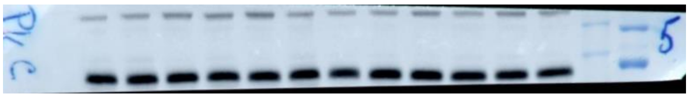

HSD HSD ACF HSD ACF ACEi HSD ACF ARB

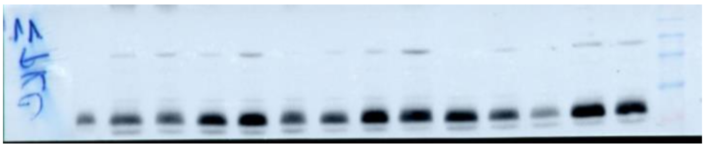

TGR HSD TGR ACF TGR ACF ACEi TGR ACF ARB

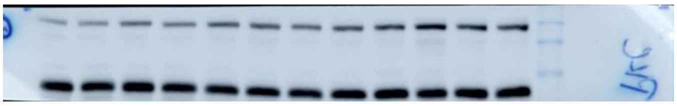

HSD HSD ACF HSD ACF ACEi HSD ACF ARB

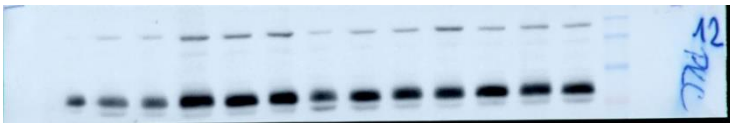

TGR HSD TGR ACF TGR ACF ACEi TGR ACF ARB

PKCe - RV

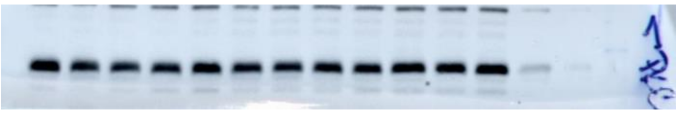

HSD HSD ACF HSD ACF ACEi HSD ACF ARB

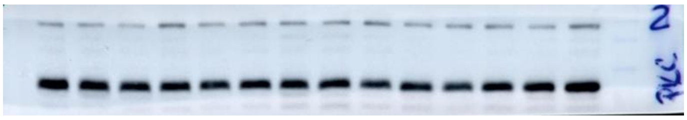

TGR HSD TGR ACF TGR ACF ACEi TGR ACF ARB

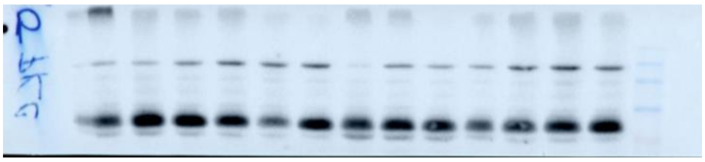

HSD HSD ACF HSD ACF ACEi HSD ACF ARB

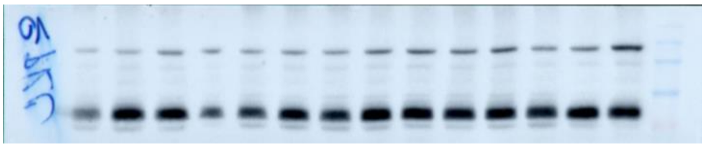

TGR HSD TGR ACF TGR ACF ACEi TGR ACF ARB

SMAD2 - LV

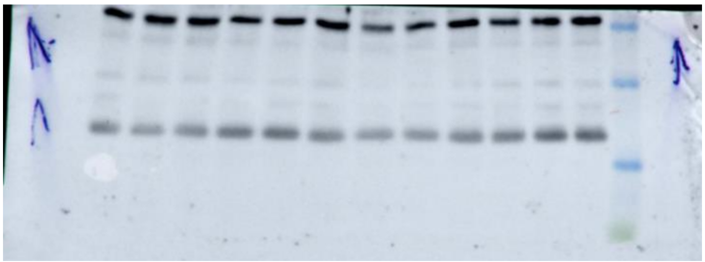

HSD HSD ACF HSD ACF ACEi HSD ACF ARB

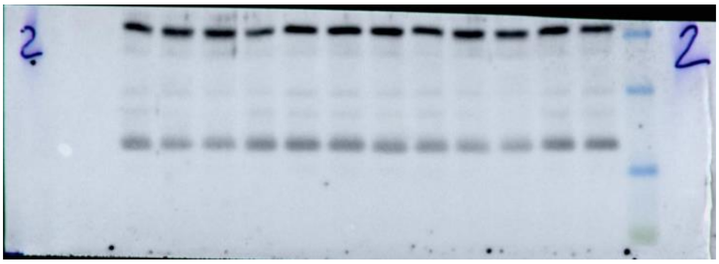

TGR HSD TGR ACF TGR ACF ACEi TGR ACF ARB

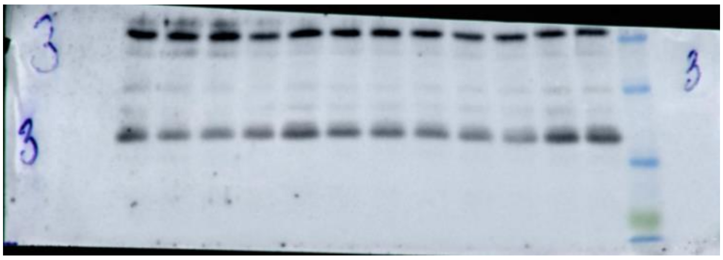

HSD HSD ACF HSD ACF ACEi HSD ACF ARB

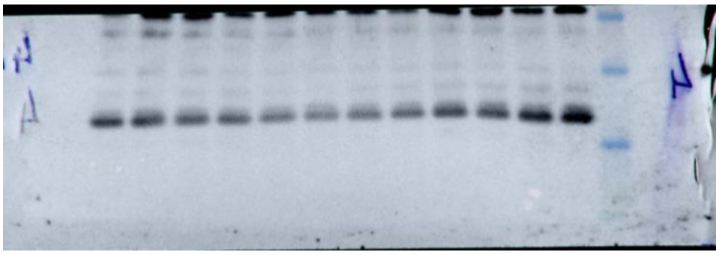

TGR HSD TGR ACF TGR ACF ACEi TGR ACF ARB

SMAD2 - RV

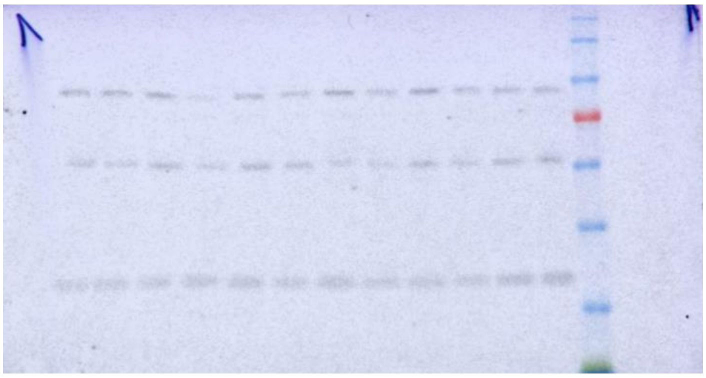

HSD HSD ACF HSD ACF ACEi HSD ACF ARB

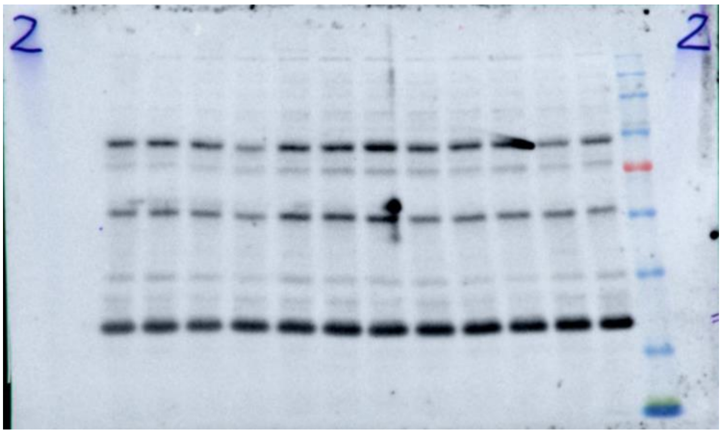

TGR HSD TGR ACF TGR ACF ACEi TGR ACF ARB

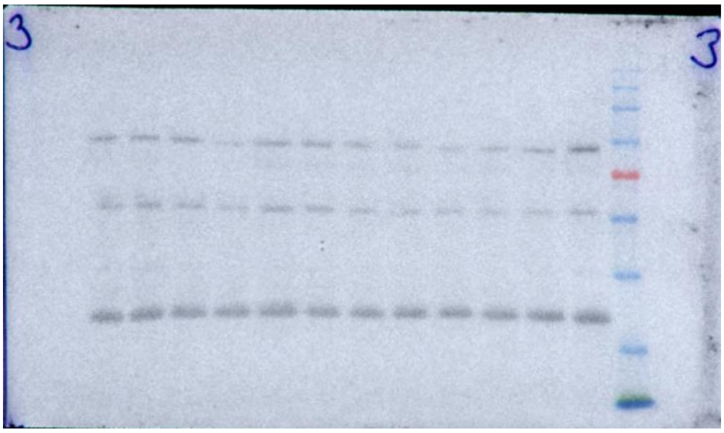

HSD HSD ACF HSD ACF ACEi HSD ACF ARB

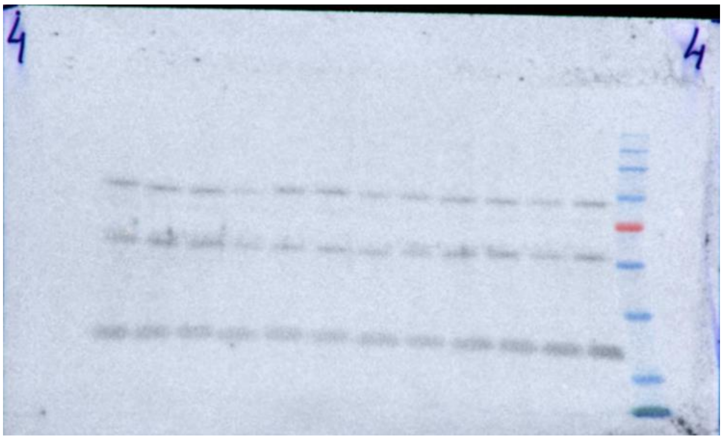

TGR HSD TGR ACF TGR ACF ACEi TGR ACF ARB

PKCd - LV

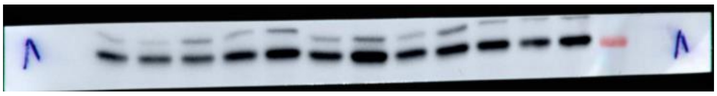

HSD HSD ACF HSD ACF ACEi HSD ACF ARB

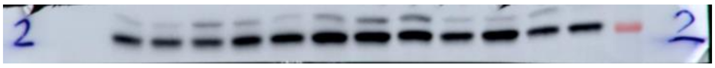

HSD TGR TGR ACF TGR ACF ACEi TGR ACF ARB

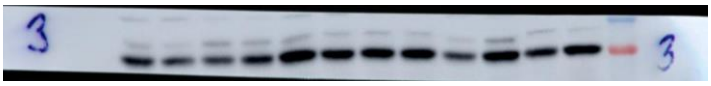

HSD HSD ACF HSD ACF ACEi HSD ACF ARB

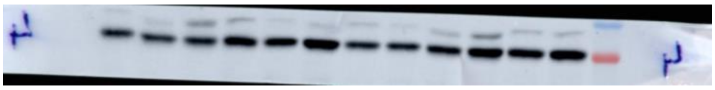

HSD TGR TGR ACF TGR ACF ACEi TGR ACF ARB

PKCd - RV

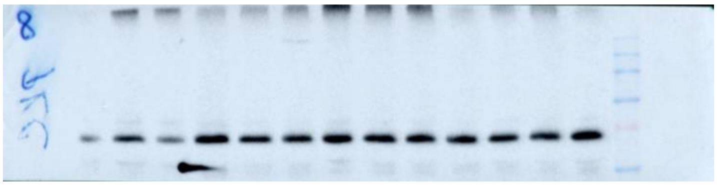

HSD HSD ACF HSD ACF ACEi HSD ACF ARB

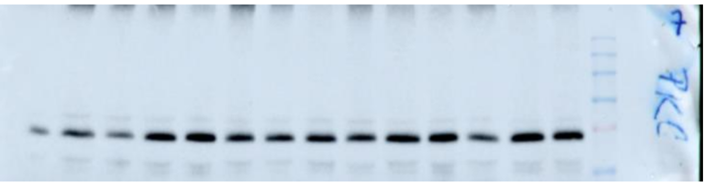

HSD TGR TGR ACF TGR ACF ACEi TGR ACF ARB

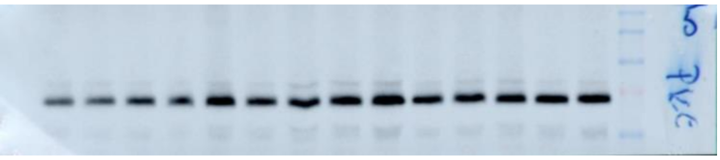

HSD HSD ACF HSD ACF ACEi HSD ACF ARB

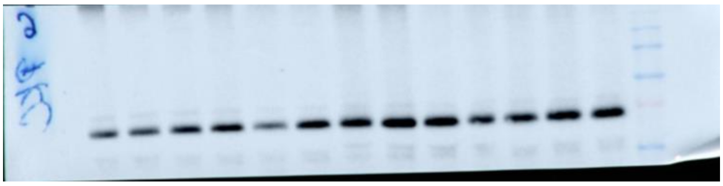

HSD TGR TGR ACF TGR ACF ACEi TGR ACF ARB

PCx43 368 - LV

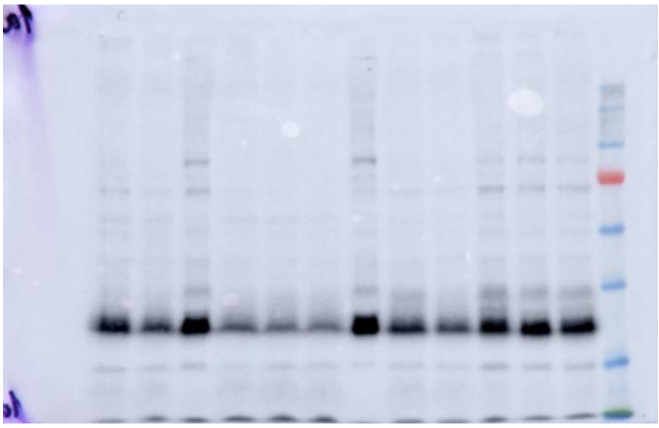

HSD HSD ACF HSD ACF ARB HSD ACF ACEi

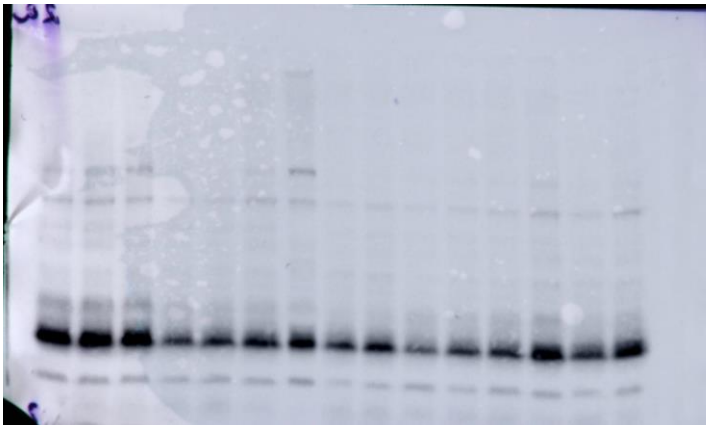

HSD TGR TGR ACF TGR ACF ARB TGR ACF ACEi

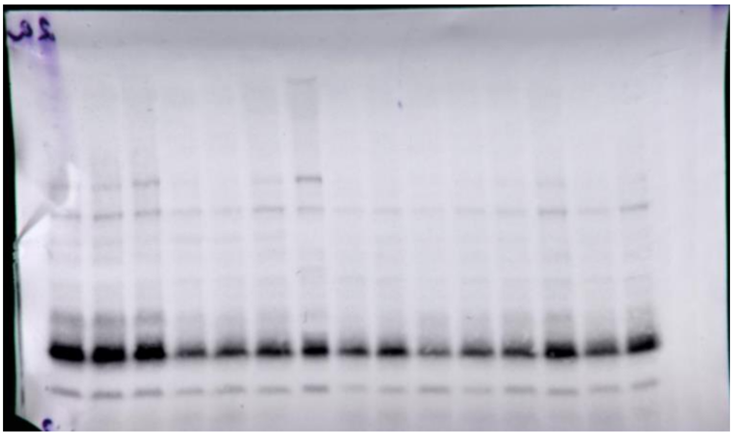

HSD TGR TGR ACF TGR ACF ARB TGR ACF ACEi

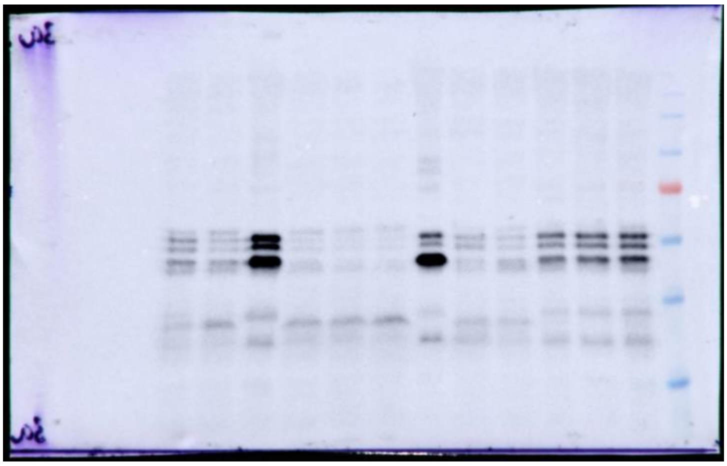

HSD HSD ACF HSD ACF ARB HSD ACF ACEi

PCx43 368 - RV

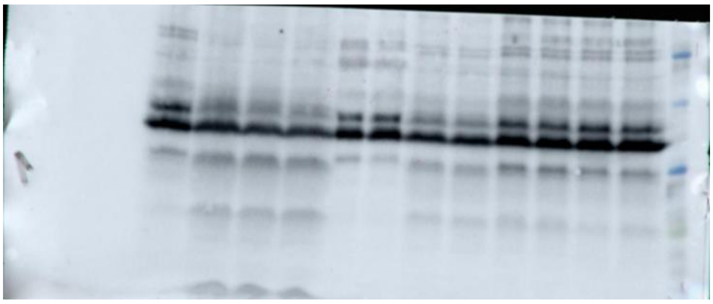

HSD HSD ACF HSD ACF ARB HSD ACF ACEi

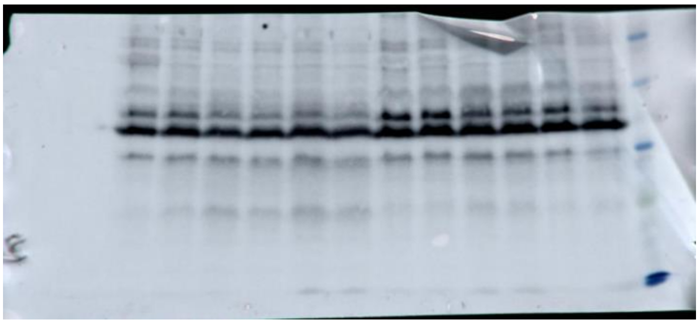

HSD TGR TGR ACF TGR ACF ARB TGR ACF ACEi

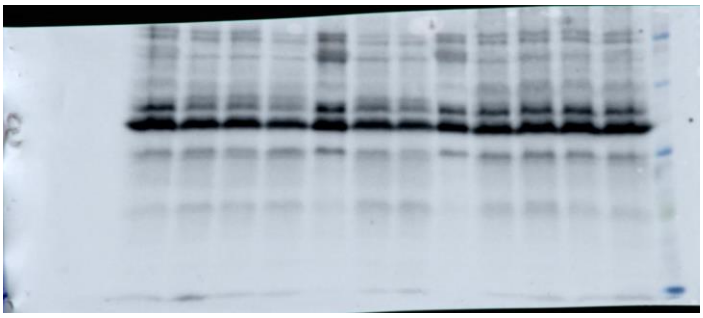

HSD HSD ACF HSD ACF ARB HSD ACF ACEi

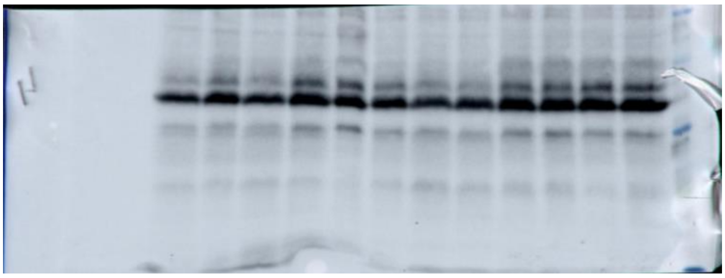

HSD TGR TGR ACF TGR ACF ARB TGR ACF ACEi

Cx43 - LV

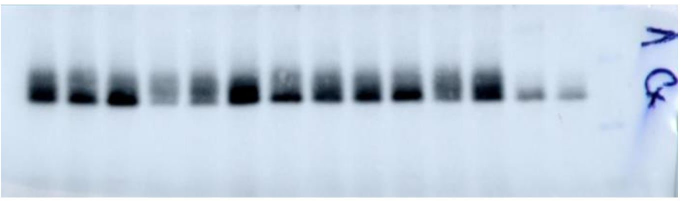

HSD HSD ACF HSD ACF ARB HSD ACF ACEi

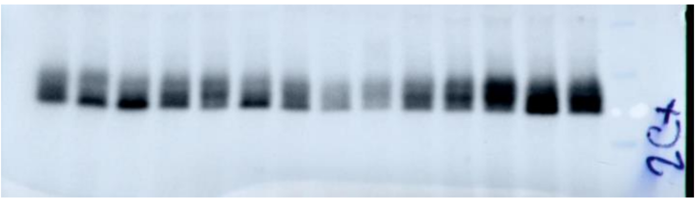

HSD TGR TGR ACF TGR ACF ARB TGR ACF ACEi

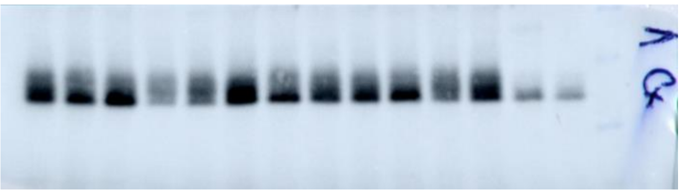

HSD HSD ACF HSD ACF ARB HSD ACF ACEi

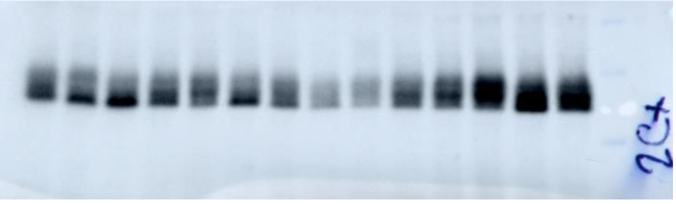

HSD TGR TGR ACF TGR ACF ARB TGR ACF ACEi

Cx43 - RV

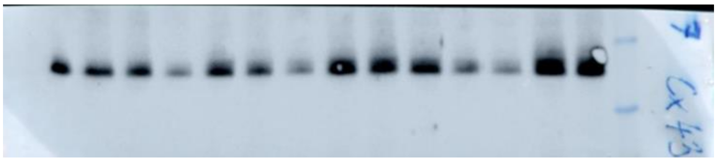

HSD HSD ACF HSD ACF ARB HSD ACF ACEi

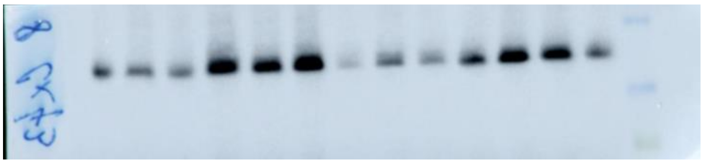

TGR HSD TGR ACF TGR ACF ARB TGR ACF ACEi

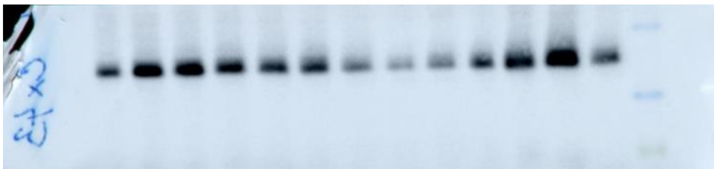

HSD HSD ACF HSD ACF ARB HSD ACF ACEi

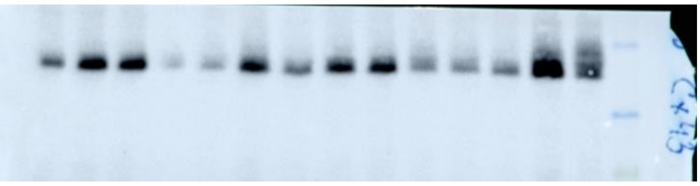

HSD TGR TGR ACF TGR ACF ARB TGR ACF ACEi

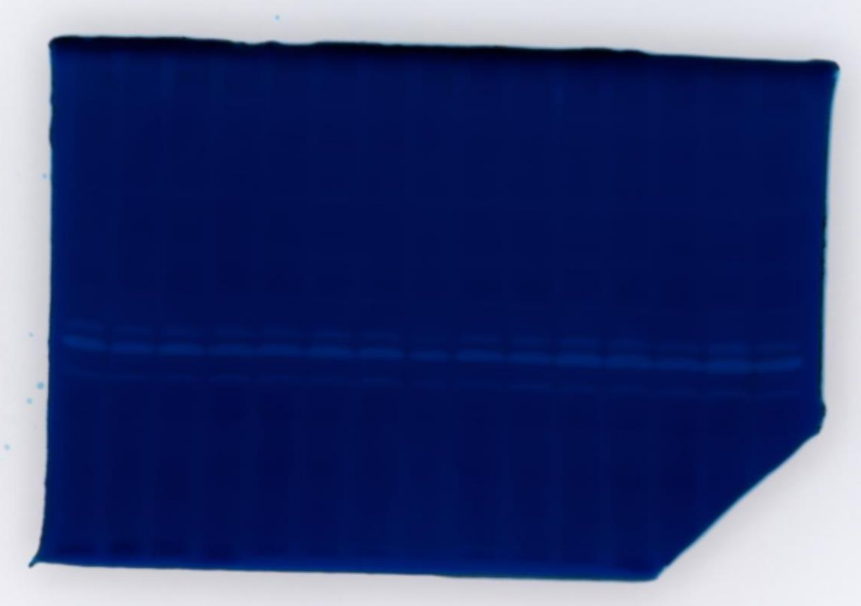

HSD HSD ACF HSD ACF ACEi HSD ACF ARB

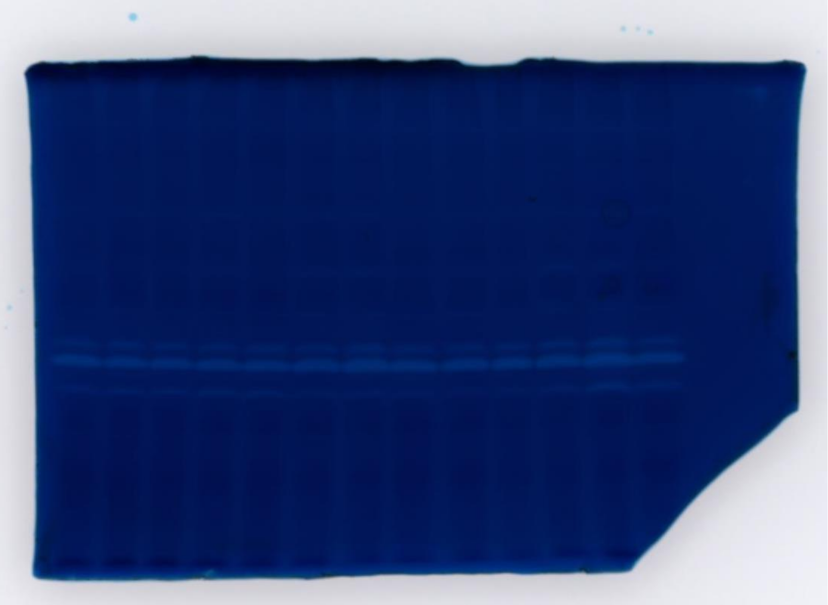

HSD TGR TGR ACF TGR ACF ACEi TGR ACF ARB

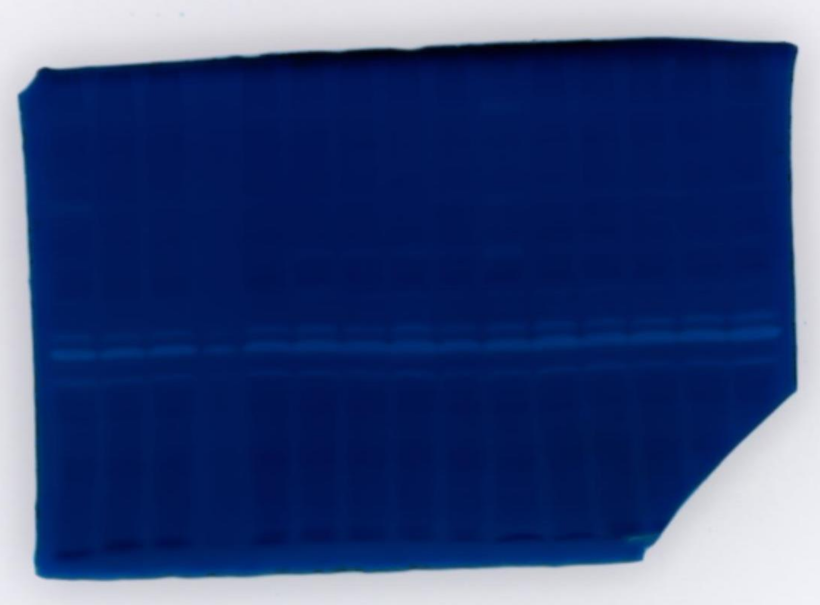

HSD HSD ACF HSD ACF ACEi HSD ACF ARB

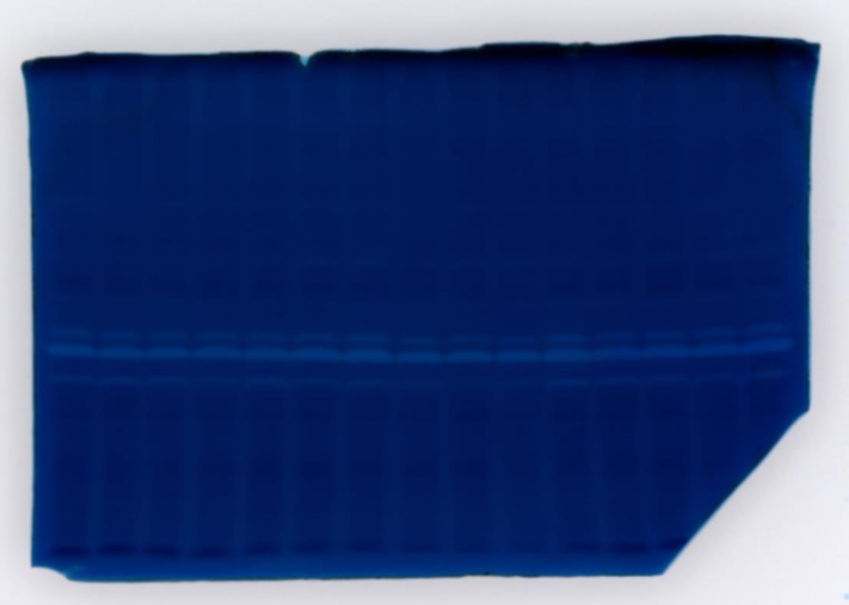

HSD TGR TGR ACF TGR ACF ACEi TGR ACF ARB
