# Supplementary material for: The treatment with trandolapril and losartan attenuates pressure and volume overload alternations of cardiac connexin-43 and extracellular matrix in Ren-2 transgenic rats
Source: Sci Rep. 2023 Nov 27;13:20923. doi: 10.1038/s41598-023-48259-2 (PMC10684879; doi:10.1038/s41598-023-48259-2)
Supplement: Supplementary file 2 — Supplementary Information 2. [file 41598_2023_48259_MOESM2_ESM.docx]

**The treatment with Trandolapril and Losartan attenuates pressure and volume overload alternations of cardiac connexin-43 and extracellular matrix in Ren-2 transgenic rats**

Matus Sykora^1^, Vojtech Kratky^2,3^, Luděk Červenka^2,4^, Libor Kopkan^2^, Narcis Tribulova^1^, Barbara Szeiffova Bacova^1^

^1^Centre of Experimental Medicine, Institute for Heart Research, Slovak Academy of Sciences, 841 04 Bratislava, Slovakia

^2^Center for Experimental Medicine, Institute for Clinical and Experimental Medicine, 140 21 Prague, Czech Republic

^3^Department of Nephrology, First Faculty of Medicine, Charles University and General University Hospital in Prague, 128 08 Prague, Czech Republic

^4^Department of Internal Medicine I, Cardiology, University Hospital Olomouc and Palacký University, Olomouc, Czech Republic

**Author for correspondence:**

Barbara Szeiffova Bacova

Centre of Experimental Medicine, Institute for Heart Research, Slovak Academy of Sciences, 841 04 Bratislava, Slovakia

Email: barbara.bacova @savba.sk

**Supplementary figure 1:**

Experimental rat groups and design of the experiment

| **Experimental group** | **HSD**(n) | **TGR**(n) |
| --- | --- | --- |
| **Sham rats** | 10 | 10 |
| **ACF** | 10 | 4* |
| **ACF + ACEi** | 10 | 10 |
| **ACF + ARB** | 10 | 10 |


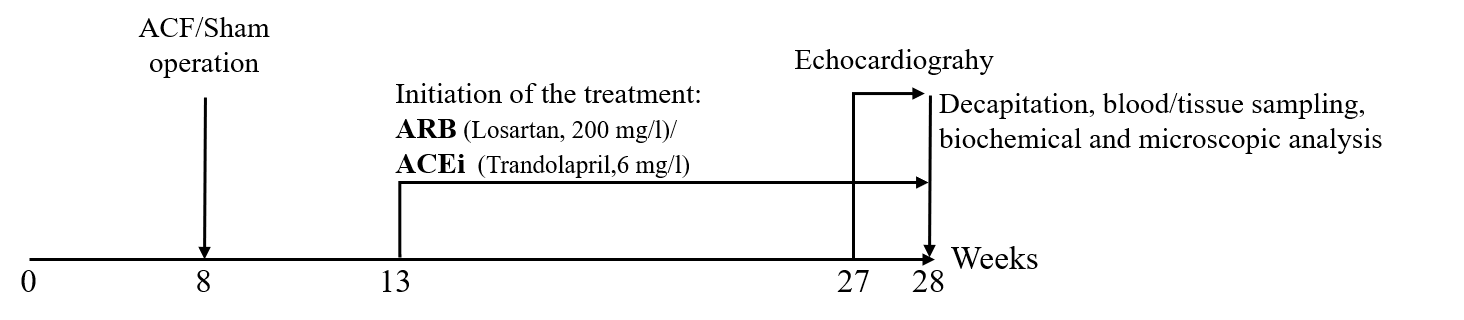


Supplementary figure 1. Experimental rat groups and design of the experiment. ACF was created in the 8 weeks old HSD and TGR rats and after five weeks from successful ACF induction, were rats treated for 15 weeks with an AT1 receptor blocker losartan (200 mg/l) or an ACE inhibitor trandolapril (6 mg/l), drugs dissolved in drinking water. HSD- Hannover Sprague- Dawley rats, TGR- hypertensive heterozygous Ren-2 transgenic (mREN2)27 rats, ACF- aortocaval fistula-induced congestive heart failure, ACEi- ACE inhibitor, ARB- AT1 receptor blocker, n-sample size, *- increased rats’ mortality in the experimental group.

**Please note, TGR ACF rats suffered with higher incidence of mortality (60%).**

**Supplementary figure 2**: Visualization of Cx43 topology


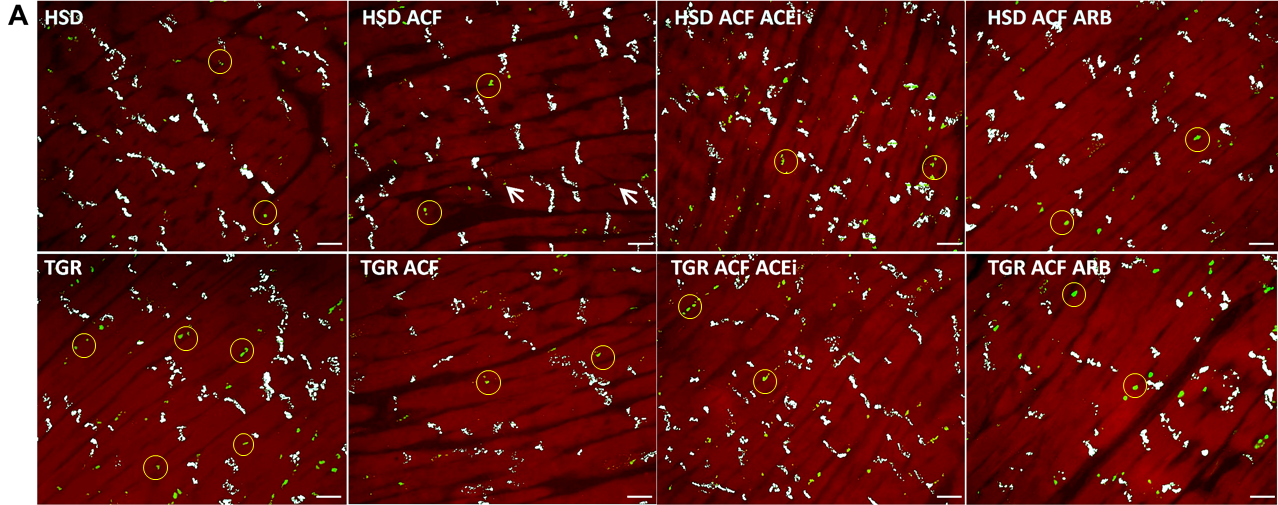


Supplementary figure 2. **A** - Visualisation of the myocardial connexin-43 using immunofluorescence staining. For better visualization of Cx43 topology, **not used for QIA**, we replaced in the representative images, the green colour of Cx43 located at the intercalated discs of the cardiomyocytes to white colour and laterally oriented Cx43 are visible in green colour. Some selected lateral topology of Cx43 is highlighted by yellow circles.

HSD – normotensive Hannover Sprague- Dawley rats; TGR – hypertensive heterozygous Ren-2 transgenic (mREN2)27 rats; ACF – aortocaval fistula; ACEi – angiotensin-converting enzyme inhibitors; ARB – Angiotensin II Receptor Blockers. Scale bar represents 200 µm. 10 µm thick frozen tissue sections from the apex of the heart were used. Results are the mean ± SD **(sample size is illustrated in supplementary figure 1). Please note, TGR ACF rats suffered with higher incidence of mortality (60%).**

**Supplementary table 1**.: Antibodies used for Western blot analysis, Antibodies used for Immunofluorescence methods

Antibodies used for Western blot analysis

| **Antibody** | **Dilution** | **Host** | **Type** | **Supplier/# Catalogue** |
| --- | --- | --- | --- | --- |
| anti-Cx43 | 1:5000 | Rabbit | Polyclonal | Sigma-Aldrich, St.Louis, MO, USA, #C6219 |
| anti-phospho-ser368-Cx43 | 1:1000 | Rabbit | Polyclonal | Santa Cruz Biotechnology, Dallas, TX, USA, #sc-101660 |
| anti-PKC-epsilon | 1:2000 | Rabbit | Polyclonal | Santa Cruz Biotechnology, Dallas, TX, USA, #sc-214 |
| anti-PKC-delta | 1:2000 | Rabbit | Polyclonal | Santa Cruz Biotechnology, Dallas, TX, USA, # sc-213 |
| anti-SMAD2/3 | 1:1000 | Rabbit | Polyclonal | Cell Signaling Technology, Danvers, MA, USA, #3102 |
| anti-Collagen I | 1:1000 | Mouse | Monoclonal | Abcam Inc.,Toronto, ON, Canada, # ab90395 |
| anti-MMP2 | 1:500 | Rabbit | Polyclonal | Santa Cruz Biotechnology, Dallas, TX, USA, # sc-10736 |
| anti-GAPDH | 1:1000 | Rabbit | Polyclonal | Santa Cruz Biotechnology, Dallas, TX, USA#sc-25778 |
| β-actin | 1:2000 | Mouse | Monoclonal | Sigma-Aldrich, Missouri, MO, USA, # A5441 |
| Anti- Rabbit | 1:2000 | - | - | Cell Signaling Technology, Danvers, MA, USA, #7074S |
| Anti-Mouse | 1:2000 | - | - | Cell Signaling Technology, Danvers, MA, USA, #7076C |

Antibodies used for Immunofluorescence methods

| **Antibody** | **Dilution** | **Host** | **Type** | **Supplier/# Catalogue** |
| --- | --- | --- | --- | --- |
| anti-Cx43 | 1:2000 | Rabbit | Polyclonal | Sigma-Aldrich, St.Louis, MO, USA, #C6219 |
| Anti-Rabbit, Alexa Fluor 594 | 1:1000 | - | - | Jackson Immuno Research Labs, West Grove, Pennsylvania, USA, #111-585-144 |
| Anti-Mouse, FITC | 1:1000 | - | - | Jackson Immuno Research Labs, West Grove, Pennsylvania, USA, #115-095-062 |
